# 

**Published:** 1887-08-20

**Authors:** 


### The Hospital.

**An Institution, Family, and Parochial Journal of Hospitals, Asylums, and all Agencies for the Care of the Sick, Criticism and News.**

SATURDAY, AUGUST 20TH, 1887.

### NOTICE TO ADVERTISERS.

THE scale of charges for small prepaid Advertisements—Situations Wanted, Situations Vacant, etc., etc., is as follows :—

| | s. | d. |
|---|---|---|
| One insertion of three lines | 1 | 0 |
| Three ,, ,, ,, | 2 | 6 |
| Per line afterwards | 0 | 6 |

*A line averages eight words.*

All copy for Advertisements must be sent to A. P. WATT, 2, PATERNOSTER SQUARE, E.C., by first post on the Tuesday *preceding* publication.

### NOTICE TO CORRESPONDENTS.

All correspondence must be written on one side of the paper only.

We cannot undertake to return MSS. not used.

**Communications,** whether intended for insertion or for private information, must be authenticated by the names and addresses of the writers, not necessarily for publication.

**Local papers** containing reports or news paragraphs **should be marked.**

It is especially requested that early intelligence of **local events of interest,** or which it may be thought desirable to bring under notice, may be sent direct to the Editor.

Communications respecting editorial matters should be addressed to the Editor, Norfolk House, Norfolk Street, Strand, London, W.C.

### THE LITERARY PRIZE.

A PRIZE of **Two Guineas** will be given every month for the best story or incident based upon hospital, or asylum, or student life, or any incident connected with the professions of medicine or nursing.

*₊* THE CHILDREN'S CORNER and AMUSEMENTS WITH PROFIT will be found on page vi of this week's issue.

### Books.

We have received the following for review :—

Churchill.
Hay Fever. By Morell Mackenzie, M.D.

Longmans.
Organic Materia Medica. By R. Bentley, M.R.C.S.
Discourses to Women. Mrs. Longshore Potts, M.D.
Pulmonary Consumption. By C. J. B. Williams, and C. T. Williams.

Simpkin, Marshall & Co.
Notes on Surgery for Nurses. By Joseph Bell.

Stock, Elliot.
The New Religio Medici. By F. Robinson, M.D.

Watt, A. P.
Forms of Nasal Obstruction. By Greville MacDonald, M.D.

EXCHANGES.

Churchill.
Journal of Mental Science, July, 1887.

Macmillan.
The Practitioner, April and May, 1887.

### QUESTIONS AND ANSWERS.

All questions should be addressed to Mr. Howard J. Collins, Secretary, The Hospitals Association, Norfolk House, Norfolk Street, Strand, W.C.

### HOME FOR LADY.

S. C.—Application may be made to Mrs. B., Belle Mere Farm, Goodby Marwood, Melton Mowbray.

### THE CHILDREN'S CORNER.

**Fourth Quarterly Prize Competition.**
Began 2nd July, 1887 ; ends 24th Sept., 1887.

**Puzzles—Eighth Week.**

No. 30. BIOGRAPHICAL ACROSTIC. 1. A celebrated Dutch admiral in the time of Cromwell. 2. A Moorish palace. 3. A gulf of the Morea. 4. An English translator of Virgil. 5. A sailing vessel. 6. The wife of Charles II. 7. A celebrated portrait painter in the seventeenth century. The initials give the name of a celebrated painter.

No. 31. CHARADE. My first is company ; my second avoids company ; my third collects company ; my whole amuses company.

No. 32 BURIED ISLANDS. 1. The car ran against the wall. 2. Wash this can, Diana. 3. He caught a chill last week.

No. 33. Why should sailors wish the Queen a long life ?

**Letters—Eighth Week.**

The letter-subject for next week will be *Butterflies.*

**Results of Sixth Week—Puzzles.**

No. 22. THE BOUQUET OF FLOWERS consisted of a Snowdrop, Dog-rose, Bluebell, and Solomon's Seal.

No. 23. DOUBLE ACROSTIC.

C abi N
R ussi A
I coniu M
C alai S
K en T
E nigm A
T u B

No. 24. HISTORICAL CHARADE. The hero is Hotspur, who was killed at the Battle of Shrewsbury.

No. 25. SQUARE WORD. The *lights* here are :

W I N G
I D E A
N E A T
G A T E

*All right*—Agatha, A. C. K. ; *Three right*—Fern, Socrates, Poodle, Toby, Le Cerf Agile, Mikado, Umslopogaas, Mount Edgcumbe, Skye, Ossian, Ellie ; *Two right*—A. G. S. McCulloch, The Eda, Scrooge, Princess Ida.

**Letters.**

As I expected, all my little friends are very fond of "fruit," and most of them have much difficulty in deciding upon their favourite kinds. Here is a letter from Mount Edgecumbe, telling us all about the banana :—

I think my favourite fruit is the banana, which only grows in very hot countries, for instance, in India or Australia. At the latter place I have seen great quantities, and also at Ceylon when coming home to England from Australia. The banana trees in growth and form are very peculiar ; they have one stem, and from this sprout huge thick green leaves, on which the banana grows. When young, the banana is a little green thing, not unlike a pear, only the same thickness at both ends, but when it gets ripe it changes its colour to a pale yellow, and it is fit to eat when you feel it is soft. This fruit grows in large bunches. I have known as many as one hundred and eighty on a bunch : and when at Colombo in Ceylon we bought a bunch containing one hundred and twenty bananas, three cocoa-nuts, and four pineapples for eighteenpence, whereas in England a single banana costs twopence. This fruit is also very useful to sick people, and doctors often advise them to take bananas. In Australia we had some banana trees, but as we lived in Tasmania we did not have full benefit of them, except once a year, when we used to have some branches sent to us.

Ossian once after a long drive in Scotland, says that he enjoyed a pocketful of haws and some mountain ash berries more than anything he had ever tasted. Ellie and Skye both much regret that the strawberries are all over, while Toby and Socrates hail with delight the advent of the apple season. Princess Ida writes :—

I like all fruits, but my favourites are apricots, cherries, and apples. Apricots are generally grown against a sunny wall, where they are protected from the wind. They are in season in August, and are a very pretty fruit, being of a yellowish colour, tinted with red, and with a lovely bloom all over them. Cherries are grown in different places, according to their kind ; some grow on trees and some against walls. These also are very pretty, they are so varied in colour, some deep crimson, some scarlet, and some yellow. They are in season in June and July. There are so many different kinds of apples that it would take me much too long to describe them all. The kind I like best are the russets, which are best in October, and sometimes November, according to the s ason. Russet-apple trees are generally large, and the apples are of a brownish colour.

Three marks each :—Toby, Mount Edgcumbe. Princess Ida, Socrates. Two marks each :—Skye, Le Cerf Agile, Ellie, Ossian.

**Notices to Correspondents.**

LE CERF AGILE.—" When others also guessed them" should have been understood.

Answers must be addressed to the " PUZZLE EDITOR," THE HOSPITAL, Norfolk House, Norfolk Street, Strand, W.C., not later than Thursday next. The " Children's Corner" Coupon (see advertisement pages) must be enclosed.

The address and full Christian name and surname, and the age of the competitor, must be stated in all replies. A *nom de plume* may be added for the purposes of publication.

### AMUSEMENTS WITH PROFIT.

### PRIZE COMPETITIONS.

**Quarterly Prize Competition.**
Began 23rd July ; ends 15th October.

**A Prize of Three Guineas**
will be awarded to the Competitor who shall have sent in the largest number of correct or best answers. No one will be permitted to win the Three Guinea Prize twice in any one year, but we shall award annually an additional

**Prize of Five Guineas**
to the competitor who shall have sent in the largest number of correct or best answers during the twelve months.

**No. 9. Double Acrostic.**

Beneath the shadow of the trees,
Where earliest sunbeams come and go,
My portly *first* in silence paces,
While afar my *second's* voice is
Heard in cadence sweet and low,
Sweet and low, and soothingly
Falls its voice on weary ears ;
While weary forms with suffering sated,
By my *whole* and hope elated,
Gain new strength for coming years.

1. On the banks of a beautiful river I stand,
For commerce and learning I'm famed in the land.
2. "Creatures of a day,"
3. Though oft opposed, of hope bereft,
Those on my side will ne'er be left.
4. A wand of office borne in state
Before a city magnate great.
5. " Blameless king and stainless man ;"
6. Source of keenest pleasure we,
Source of poignant misery.

**No. 10. Arithmetical Puzzle.**

A party of fifteen friends started on a walking tour of seven days' duration. They made daily excursions in parties of three, but the same three persons never walked twice together. How did they arrange their parties ?

**Answers.**

No. 5. DOUBLE ACROSTIC.

V agabon D
I slande R
R a Y
G oa D
I r E
L iberatio N

Correct answers have been received from Gretta, Esau, Ostrich, Condorcet, V. W.

**No. 6. Rhythmical Recreation.**

The definitions of the "*Healing Art*" are exceedingly good, and I regret that want of space will only allow of one answer being inserted here.

" When, smitten sore with varied pain,
Crowds press for healing, ever new,
How blest the throng made well again,
Which late distressful met our view.
All blessings on the heaven-sent art,
Which heals sore hurt or trivial smart."

BROWNIE.

Answers have been received from Brownie, Ellice, Ostrich, V. W., Condorcet, Aralc, Gretta, Mater, Perseverance, Man of the Sea.

**Notices to Correspondents.**

GRETTA.—Under these circumstances it is not necessary.

Answers must be addressed to THE PRIZE EDITOR, AMUSEMENTS WITH PROFIT, THE HOSPITAL, Norfolk House, Norfolk Street, Strand, W.C., not later than the *first* post on Friday next. The full name and address must in all cases be given, but a *nom de plume* may be added for publication. Only one side of the paper should be written on. The " Amusements with Profit" Coupon (see advertisement pages) must be enclosed with replies.

